# Editorial: Development of the precision diagnostics and treatment for duchenne/becker muscular dystrophy

**DOI:** 10.3389/fneur.2024.1396816

**Published:** 2024-03-22

**Authors:** Corrado Italo Angelini

**Affiliations:** Department of Neuroscience, School of Medicine and Surgery, University of Padua, Padua, Veneto, Italy

**Keywords:** duchenne dystrophy, Becker dystrophy, caregiver, overload, quality of life, therapy

Dystrophinopathies are a rather heterogeneous group of rare X-linked autosomal disorders both in progression and clinical manifestations, characterized by two phenotypes a severe phenotype or Duchenne muscular dystrophy (DMD) and a milder phenotype named Becker muscular dystrophy (BMD).

DMD and often also Becker dystrophy are associated with the initial stages of cardiomyopathy, due to the dystrophic involvement of the heart muscle. As the disease progresses, the heart muscle becomes weaker, leading to potential cardiac complications such as heart failure and arrhythmias. These cardiac issues can significantly impact the quality of life in individuals as well as DMD carriers of this gene mutation, which might present a series of cardiac and skeletal muscle changes, dependent on the degree of X-linked inactivation, according to the Lyon hypothesis.

The topic “*Developing precision diagnosis and treatment for Duchenne/Becker muscular dystrophy*” might involve a multi-faceted approach that integrates cutting-edge technologies, personalized medicine, and advancements in genetic and molecular research.

Some key strategies pertinent to the topic are accurate genetic testing which is crucial for diagnosing DMD/BMD. Profiles and disease characteristics can enhance the efficacy of therapies for Duchenne/Becker muscular dystrophy. Personalized medicine approaches can include gene therapy, and exon-skipping drugs, to target specific genetic mutations. Clinical trials and treatment guidelines pertain to the topic since engaging in targeted clinical trials that focus on precision medicine interventions can facilitate the development of novel therapies for Duchenne/Becker muscular dystrophy.

In the Research Topic to develop the above aims, a response was given by four different world areas that submitted relevant articles contributing variably to the topic.

Juríková et al. have focused on the low quality of life in Duchenne dystrophy studying a series of DMD cases, DMD patients were of various ages and exhibited different degrees of disability in functional neurological and cardiac impairment. The most important results of this research study were the relation/association between better movement of the upper limbs and total mobility, self- service, and good/bad days in patients.

Decreased QoL and movement of the upper limbs were studied by a questionnaire on the disease that was completed both by DMD boys and their parents. In DMD the quality of life can indeed be significantly impacted by various factors, including cardiac function. Juríková et al. found that DMD boys taking ACE inhibitors/sartans had better overall mobility than DMD who were without therapy for heart management. Maintaining good heart function through cardiac monitoring, medications, and interventions is crucial in managing DMD and improving QoL. Regular cardiac assessments, including echocardiograms and electrocardiograms, can help in detecting and managing heart-related complications early. Additionally, supportive care, physical therapy, respiratory support, and regular follow-ups with healthcare providers specializing in DMD can also contribute to enhancing the overall QoL for DMD boys. Both DMDs and their caregivers need to work closely with a multidisciplinary healthcare team to address both the skeletal muscle and cardiac aspects of the disease to optimize outcomes and QoL.

Malaga et al. reviewed treatments available for DMD in a scoping review. The guidelines provided some recommendations for pharmacological treatment with rather broad aims including recommendations for treatment with steroids. They recommended against the use of ataluren to reduce mortality, improve quality of life, and reduce dyspnea and fatigue. However, the NICE guideline evaluated the application of ataluren in DMD boys 2 years and over, who can walk and gave a conditional recommendation in the context of a managed access agreement and other financial components to reduce the total costs, although recently EMEA has suspended ataluren's use due to lack of proven efficacy. Additionally, the guidelines presented agree on the use of prednisone or deflazacort to reduce mortality, improve timed motor functions, and reduce the progression of scoliosis. New drugs are on the way, such as the new steroid vamorolone for both DMD/BMD.

Fortunato et al. contributed with a review of clinical and diagnostic characteristics of BMD, regarding in-frame deletions of dystrophin molecules and they proposed that preservation of Hinge 1 and 2 domains of dystrophin might be sufficient to keep an almost normal muscle function. DMD gene therapy is already viable in several clinical trials, provisionally approved by the FDA for several types of gene constructs. The full conservation of H1 and H2 domains in Becker asymptomatic individuals in their cohort might suggest that these domains are crucial for correct muscle function via the dystrophin interactions at the sarcolemma. The three mini-dystrophin constructs used for gene therapy are different in terms of exon content: the Sarepta construct includes H1, H2, and H4 domains, while other constructs do not.

In general, most clinical trials aim to transform the severe DMD phenotype into a milder BMD.BMD has varied clinical phenotypes, according to previous series published (1) Becker muscular dystrophy shows significantly high serum CK levels, frequently exceeding 1,000 U/l. histopathological and electromyography studies primarily reveal myopathic changes that correlate with several different dystrophin molecules of reduced quantity.

However, in BMD the initial symptoms and progression differ significantly. Approximately 2% of patients presented with limb weakness similar to DMD patients but at an older age (intermediate phenotype). Interestingly, a considerable percentage of patients presented with symptoms not classically associated with BMD, such as muscle pain (myalgia), high CK levels in the blood (CKemia), muscle breakdown products in urine (myoglobinuria), severe reaction to certain anesthetics (malignant hyperthermia), or ankle tendon contractures. While mild weakness was identified in some of these patients, most were otherwise asymptomatic.

The social interactive aim of caregiver overload ascertainment in caregivers of neuromuscular patients was entertained by Rodriguez et al. and the group of Deusto University in collaboration with the patient Association from Mexico. They investigated the overload of caregivers of several disorders including DMD and BMD.

Regarding overload, they considered that parents of children with neuromuscular diseases (NMD) experience multiple difficulties in their daily lives that affect their physical and psychological health and the risk factors for these health issues have so far not been sufficiently investigated.

Therefore, their study aimed to analyze the potential predictors of overload in NMD parents, including QoL, somatic symptomatology, life satisfaction, psychological adjustment, and sociodemographic variables. Overload in carriers of NMD can have significant economic and social impacts on both the carriers and society as a whole. Some key points to consider are economic impact and financial burden.

Caring for NMD can be financially draining due to the costs associated with medical treatment, equipment, medications, and rehabilitation services. Caregivers may have to take time off work or reduce their working hours to care for their loved ones, leading to a loss of income and potential career advancement opportunities. The need for specialized healthcare services and frequent hospital visits can contribute to higher healthcare costs.

Social Impact and emotional and physical strains are common in NMD caregivers. The constant demands of caregiving can lead to emotional stress, physical exhaustion, and burnout, affecting their overall wellbeing and quality of life. Caregivers may experience social isolation as they may have limited time for social activities or find it challenging to maintain relationships outside of caregiving responsibilities. The growing number of neuromuscular patients and their caregivers may increase the demand for support services, including respiratory care, counseling, and community resources, putting pressure on social support systems. One of the most relevant findings of the study is the identification of three overload groups based on life satisfaction and somatic symptom scores within the predictive model of the discriminate analysis. In the literature, somatic symptoms have been associated with the likelihood of caregiving stress and mood disorders. According to the hypothesis proposed by Rodriguez et al. somatic symptoms, fatigue, and sleep disturbances have been associated with increased caregiver overload. Regarding life satisfaction and overload, the mechanisms underlying may be that life satisfaction is produced by the positive aspects of caregiving. If caregivers find positive aspects they may improve their life satisfaction and might not suffer from overload despite having many caregiving tasks. Positive aspects of caregiving can lead to a higher perception of reward and increased self-esteem.

Addressing the overload experienced by carriers of NMD requires a comprehensive approach involving healthcare providers, policymakers, employers, and community support networks. Providing adequate support, resources, and services can help mitigate the economic and social impact and improve the overall wellbeing of both the patients and their caregivers, whose QoL is aggravated by these issues. Compared to other studies, such as the one by Kanters et al. (2) who studied Pompe disease parents in Rodriguez et al. study caregivers had more problems in activities of daily living, more physical problems, and emotional distress. However, it was also found that almost all of them reported at least some “satisfaction” in providing care and that they received support from others in carrying out their caregiving tasks since the caregiver experience can sometimes be satisfying. In a group of 502 DMD, BMD, or LGMDs key relatives, Magliano et al. (3) reported that, despite the difficulties associated with caregiving, relatives identify valuable benefits in their experience. Patient associations and support by caregivers that empower patients and families with information, resources, and support networks can enhance their understanding of the disease and treatment options for DMD/BMD. Patient advocacy groups can also drive awareness, research funding, and policy initiatives to advance precision medicine efforts. Multidisciplinary care providing comprehensive care through a multidisciplinary team of specialists, including neurologists, cardiologists, physical therapists, and genetic counselors, can optimize the management of DMD/BMD. Coordinated care plans can address the diverse needs of patients and improve outcomes. This Research Topic highlights that by employing these strategies and leveraging advancements in genetic research, molecular biology, and personalized medicine, the field can make significant progress in developing precision diagnosis and treatment options for DMD/BMD, ultimately improving patient outcomes and quality of life.

## Author contributions

CA: Conceptualization, Writing – original draft, Writing – review & editing.

## References

[1] Hoffman EP Kunkel LM Angelini C Clarke A Johnson M Harris JB. Improved diagnosis of Becker muscular dystrophy by dystrophin testing. Neurology. (1989) 39:1011–7. 10.1212/WNL.39.8.1011 2668783

[2] Kanters TA van der Ploeg AT Brouwer WB Hakkaart L. The impact of informal care for patients with Pompe disease: an application of the CarerQol instrument. Mol Genet Metab. (2013) 110:281–6. 10.1016/j.ymgme.2013.07.020 23973269

[3] Magliano L Scutifero M Patalano M Sagliocchi A Zaccaro A Civati F. Integrated care of muscular dystrophies in Italy. Part 2. Psychological treatments, social and welfare support, and financial costs. Acta Myol. (2017) 36:41–5. 28781515 PMC5530600

